# Case Ascertainment of Measles during a Large Outbreak—Laboratory Compared to Epidemiological Confirmation

**DOI:** 10.3390/diagnostics14090943

**Published:** 2024-04-30

**Authors:** Chen Stein-Zamir, Nitza Abramson, Irina Sokolov, Lia Mor-Shimshi, Hanna Shoob

**Affiliations:** 1 Jerusalem District Health Office, Ministry of Health, Jerusalem 9134302, Israel; nitza.abramson@lbjr.health.gov.il (N.A.); irina.sokolov@lbjr.health.gov.il (I.S.); lia.mor-shimshi@lbjr.health.gov.il (L.M.-S.); hanna.shoob@lbjr.health.gov.il (H.S.); 2 Faculty of Medicine, Hadassah Braun School of Public Health and Community Medicine, The Hebrew University of Jerusalem, Jerusalem 9112102, Israel

**Keywords:** measles, measles diagnosis, measles vaccine, children

## Abstract

Measles is a highly contagious viral disease, and hence, sufficient herd immunity is obligatory to prevent infection transmission. Measles is still a cause of considerable disease burden globally, mainly in children. During a national measles outbreak in Israel in 2018–2019, the peak incidence rates occurred in the Jerusalem district. Most measles cases in the Jerusalem district (75.5%, 1702) were observed in children younger than 15 years of age, 49.2% (1109) were in children under 5 years of age, and 18.9% (425) were in infants under 1 year of age. The routine measles vaccination schedule includes two doses at 1 and 6 years of age. Most cases (1828, 81.1%) were unvaccinated (zero measles vaccine doses). These cases comprised the 425 affected infants under 1 year of age, who were ineligible for vaccination, along with the 1403 children over 1 year of age, who were otherwise unvaccinated. This study aimed to describe the epidemiologic and laboratory features of this measles outbreak, and to investigate case ascertainment (laboratory confirmed compared to epidemiologically confirmed cases). The study population included 2254 measles cases notified during the period spanning June 2018 to May 2019 in the Jerusalem district (incidence rate 176 per 10,000 population). Of the 2254 cases, 716 (31.8%) were laboratory confirmed, and 1538 (68.2%) were confirmed as epidemiologically linked. Most laboratory confirmed cases (420, 58.7%) underwent real-time PCR tests. Serological tests (measles IgM and IgG) were used in 189 (26.4%) cases, and a combination of RT-PCR and serology was used in 107 (14.9%) cases. In a multivariate model analysis, the variables significantly associated (after adjustment) with higher odds for laboratory confirmation included month of disease onset (late), additional measles cases in the household (single case), place of medical treatment (hospital; either emergency department, or hospitalization) and vaccination status (at least one prior vaccine dose). The measles outbreak described demonstrates the urgency of addressing vaccination gaps with appropriate outbreak prevention programs. The road to measles elimination needs to be paved with robust public health infrastructure, excellent field epidemiology for outbreak surveillance, investigation, and control, and laboratory proficiency.

## 1. Introduction

Measles is a highly contagious, vaccine-preventable disease; however, it still contributes substantially to the global burden of disease. A high population immunity level is required to prevent infection transmission [1,2,3]. According to the Worldwide Progress Report towards measles elimination (2000–2022), none of the six World Health Organization regions has achieved and sustained measles elimination [1]. Elimination was not achieved in 102/194 (52.6%) countries, 83 countries (42.8%) reported elimination, and 9 countries re-established elimination [1]. The measles virus (Rubeola), an RNA virus, is a member of the *Morbillivirus* genus of the *Paramyxoviridae* family [4]. Measles is one of the most contagious infectious diseases. The estimated basic reproductive number (R_0_) of measles is high (R_0_ range of 12–18), reflecting the mean number of secondary infection cases arising from a measles case in a completely susceptible population [5,6]. The measles virus is transmitted by direct contact with infectious respiratory droplets and by airborne spread of small particle aerosols [6]. The incubation period of measles is 10–14 days, and cases are considered contagious for 4 days before the onset of rash until 4 days after its appearance [6]. As measles infection is extremely transmissible, it is imperative to sustain high vaccination coverage rates of 95% and above in the population [1,2,3].

Over a decade ago, the WHO set a global measles elimination goal; yet, elimination has not been achieved to date [1,2,3,7]. Measles elimination is considered feasible due to several factors. Firstly, humans are the sole known natural measles virus reservoir. Secondly, measles is a vaccine preventable disease (VPD), with effective measles-containing vaccines (MCV) having been offered for decades. Finally, diagnostic measures for the prompt detection of measles and the provision of control measures are also available [7,8,9]. The coordinated global initiatives aimed to increase measles vaccination coverage in young children have been effective, and between 2000 and 2017, the global annual incidence of measles cases showed a noticeable decline of about 83% [10,11]. During the years 2017–2019 this trend changed; multiple measles outbreaks emerged in many countries, leading to a sharp increase in the global incidence of measles [2,7,10,11]. In 2020, reported measles incidence rates declined worldwide. This can probably be attributed to the range of infection control measures that were being employed with the aim of containing the COVID-19 pandemic [10,12]. However, during the peak COVID-19 epidemic years (2020–2021), global routine childhood vaccination coverage (including measles vaccines) declined markedly [10,12]. Consequently, in 2022, the estimated number of measles cases increased by 18% globally; moreover, measles mortality increased by 43% compared to 2021 [1].

The measles control challenge necessitates ongoing sustainable prevention activities at the local, regional, national, and international levels [13]. Public health agencies utilize the international guidelines regarding protocols for conducting measles epidemiological surveillance and outbreak investigation and response [13]. The case definition for measles surveillance is a set of uniform criteria (clinical, laboratory, and epidemiological) for defining the disease [14,15]. In measles outbreak settings, prompt identification of cases with laboratory and/or epidemiological confirmation, along with the implementation of proper infection control measures, are vital to prevent further transmission [2,13]. Measles laboratory analysis includes specific serological tests of IgM and IgG antibodies, virus detection using reverse transcription (RT)-PCR tests, and genetic characterization of measles virus strains [16].

Concomitant with outbreaks in other countries, in 2018–2019, a large measles outbreak emerged in Israel (4300 notified measles cases) [17,18,19,20,21,22]. The outbreak source has been traced to measles importation events [17,18,19,20,21,22]. Measles importation from countries with active virus circulation led to subsequent spread in under-vaccinated groups [19]. The public health teams carried out investigations, case ascertainment, and control measures [17,18,19,20,21,22]. Based on a community-oriented approach, the control measures included mass vaccination campaigns in community clinics and mobile units, and the provisioning of culturally appropriate vaccine information [17,18,19,20,21,22]. About half of the measles cases notified nationally were in the Jerusalem district (*n* = 2254), whose population (1.3 million) comprises 14% of the national population [17,18,19,20,21,22]. Communities with inadequate completeness and timeliness of childhood vaccinations showed the highest incidence rates [17,18,19,20,21,22]. The Jerusalem district overall incidence rate of measles was 176 per 100,000 population, with the peak incidence rate (1174 per 100,000) being observed in infants under one year of age [17]. The disease burden was substantial, with 8% of measles patients requiring hospitalization due to reported measles complications. Additionally, two deaths occurred: an 18-month-old unvaccinated toddler, and an 82-year-old immunocompromised patient [17,19].

In light of the considerable disease burden posed by measles, the aim of this study is to investigate and describe the case ascertainment of measles during the outbreak through the assessment of laboratory confirmed measles cases compared to epidemiologically confirmed measles cases.

## 2. Methods

Measles is a notifiable disease by law in Israel; physicians and microbiological laboratories are required to notify measles cases to the local district health office [18]. The public health teams at the district health office perform epidemiological investigations using structured questionnaires. The variables collected in the epidemiological investigation include demographic characteristics, date of disease onset, case ascertainment (laboratory confirmed or epidemiologically linked), clinical manifestations in accordance with the clinical case definition, measles complications and hospitalization, and vaccination status (measles vaccine doses received prior to the outbreak). The clinical case definition of measles includes fever of 38.3 °C or greater, generalized maculopapular rash for at least 3 days, and cough, coryza, or conjunctivitis [14]. Hospitals also notify the district health office of measles-related hospitalizations [19]. The public health teams assemble additional data on measles cases from community clinics and hospital records, as applicable.

The laboratory confirmation methods used were real-time PCR tests (RT-PCR) for measles virus detection in either urine specimens or throat swabs [23,24], and measles IgM and IgG serological tests utilizing enzyme-linked immunosorbent assay (ELISA) commercial kits [25]. According to the standard recommendations, clinicians and public health teams collect the clinical samples for measles RT-PCR testing and/or serum samples for serological tests from patients as soon as possible after the onset of rash [2]. All measles laboratory tests are provided free of charge. The National Center for Measles, Mumps, and Rubella performs measles virus genotyping according to the WHO standard protocols. In the 2018–2019 measles outbreak, the variant was defined as a genotype D8 virus with sequences identical to the named strain MVs/Gir Somnath. IND/42.16, which prevailed simultaneously in the European region [26,27,28].

Upon case investigation completion, the public health teams applied measles case definition and case ascertainment criteria consistent with the WHO guidelines from the measles outbreak toolbox [13,15]. The definition of an epidemiologically linked measles case was a clinical case unconfirmed by a laboratory test, geographically and temporally linked, with dates of rash onset 7–21 days apart from a laboratory-confirmed case or another epidemiologically linked measles case [15]. Following epidemiological investigations and laboratory tests, the public health teams defined cases as discarded if they did not meet the definition for laboratory confirmation or epidemiological linkage [15].

As part of the epidemiologic investigation, vaccination status was confirmed against the national immunization registry database [29,30]. Israel adopted a two-dose measles vaccine schedule in 1990. This schedule includes two measles vaccine doses at the ages of one year and six years (in the first school grade). These vaccines are given as combination vaccines: either the MMR (measles, mumps, and rubella vaccine) or MMRV (measles, mumps, rubella, and varicella vaccine). The overall national measles vaccination coverage is reported as 95%, yet under-vaccinated groups exist, mainly in ultra-orthodox Jewish communities [18,20,25,29].

The study design was a retrospective cohort study including all notified measles cases that occurred between June 2018 and May 2019 in the Jerusalem district (*n* = 2254), with further sub-groups analysis also conducted.

Data analysis was performed using SPSS statistical software (Version 25.0, IBM Corp., Armonk, NY, USA). The statistical tests used included the Pearson Chi-square test, Fisher exact test, non-parametric tests, and odds ratio (OR) with 95% confidence interval (CI). A *p* value of less than 0.05 was considered significant for all comparisons. After descriptive assessment, a comparison analysis was performed between the two case ascertainment groups: laboratory confirmed cases and epidemiologically linked cases. The two group comparison of proportions was performed using the Chi-square test, Fisher exact test, and OR with 95% CI, and the comparison of medians was elicited using the Wilcoxon–Mann–Whitney U two-sample test. Additionally, the types of laboratory methods employed (RT-PCR, serological IgM and IgG tests, and combination) were assessed in the laboratory confirmed group. A multiple logistic regression analysis model was formulated for the dependent variable. It was defined as “measles laboratory confirmation”, and adjustments were made for the following variables: case age, gender, date of disease onset, presence of additional measles cases in the household, place of medical treatment (community clinic, emergency department or hospitalization), measles vaccination status, and place of residence.

The study was approved by the Institutional Review Board of the Ministry of Health, Israel. All collected and analyzed data were processed anonymously and in confidentiality, in strict observance of legislation and guidelines on observational studies.

## 3. Results

The study population included 2254 measles cases notified from June 2018 to May 2019 in the Jerusalem district. Of the 2254 cases, 716 (31.8%) were laboratory confirmed cases and 1538 (68.2%) were epidemiologically linked. The general characteristics of the measles cases are presented in Table 1. Most cases (75.5%, 1702) were in children younger than 15 years of age, and 49.2% (1109) were in children under 5 years of age. The two study groups differed in several characteristics. The laboratory confirmed cases (median age 9.2 years) were older than the epidemiologically linked cases (median age 4.6 years). Infants under 1 year of age (*n* = 425) comprised 18.9% of the cases. Most infants were aged 6–12 months (360/425, 84.7%); 51.5% (219/425) were aged 9–12 months. Males were overrepresented, mainly in the laboratory confirmed cases (62.2%). The case ascertainment method was associated with measles cases in the household (a single case *n* = 1172, two cases and more *n* = 1082). A higher proportion of households with a single measles case was observed in laboratory confirmed measles cases (72.5%) compared to epidemiologically linked cases (42.5%).

**Figure 1:**
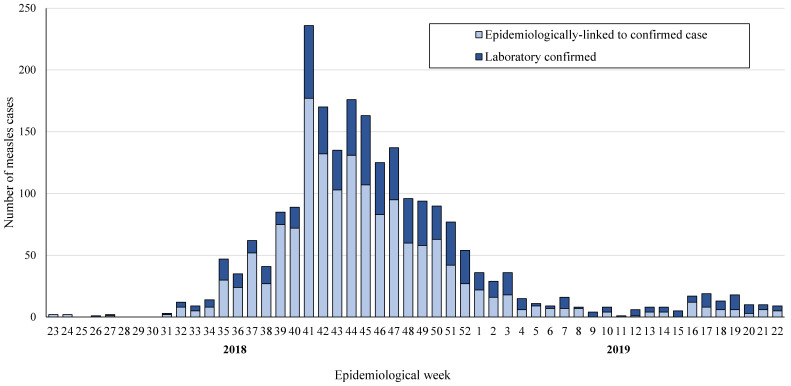
The number of notified measles cases (*n* = 2254) and the relative proportions of the laboratory confirmed cases (*n* = 716) and the epidemiologically linked cases (*n* = 1538), by epidemiological week, from the 23rd week of 2018 to the 22nd week of 2019 in the Jerusalem district, Israel.

The group proportions were associated with the outbreak phase. Figure 1 presents the relative proportions of laboratory confirmed and epidemiologically linked cases in the Jerusalem district, by epidemiological week (from week 23 of 2018 to week 22 of 2019). The measles outbreak peaked during weeks 40–48 of 2018. Overall, about half (46%) of the measles cases (*n* = 1037) were notified between June 2018 and October 2018, and 54% of cases (*n* = 1217) were notified between November 2018 and May 2019 (Table 1, Figure 1). The odds of laboratory ascertainment of measles cases were significantly higher in the second half of the measles outbreak in the Jerusalem district (from November 2018 to May 2019) compared to the first half (from June 2018 to October 2018) of the outbreak (OR = 1.99, 95% CI 1.65–2.39, *p* = 0.0001).

**Figure 2:**
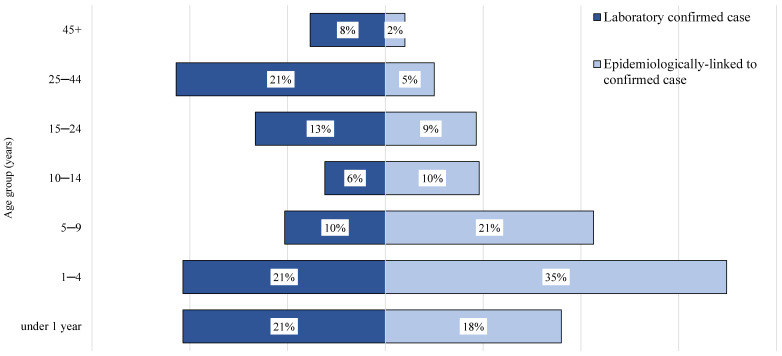
The distributions across seven age groups of the measles cases in the two study groups: laboratory confirmed and epidemiologically linked, measles outbreak 2018–2019, Jerusalem district, Israel.

The age groups distribution of measles cases in the two study groups is presented as a horizontal bar graph in Figure 2. The age groups were under 1 year, 1–4 years, 5–9 years, 10–14 years, 15–24 years, 25–44 years, and 45 years and above. The age groups distribution differed, with 74% of the epidemiologically linked cases being in the age groups younger than 10 years of age, compared to 52% of the laboratory confirmed measles cases (OR = 2.72, 95% CI 2.24–3.28, *p* = 0.0001).

The clinical signs of measles, place of provision of medical treatment, and the vaccination status of all cases and the two study groups are presented in Table 2. The groups differed as to the prevalence of classic clinical signs of measles, with fever, coryza, and conjunctivitis being more prevalent among epidemiologically linked cases. Measles complications, including pneumonia and otitis, were more prevalent among laboratory confirmed cases. Regarding the place of medical treatment, most cases (1849, 82%) were treated in community clinics, 9.5% (215 cases) visited the hospital emergency department, and 8.4% (190 cases) were hospitalized. The place of medical treatment differed between the groups; laboratory confirmed measles cases were more likely to visit the hospital emergency department and to be treated in hospitals, compared to epidemiologically linked measles cases (35.6% vs. 9.8%, OR = 5.1, 95% CI 4.05–6.47, *p* = 0.0001).

Regarding vaccination status, most of the observed measles cases (1828, 81.1%) were unvaccinated. Only 9.8% (220 cases) had received one measles vaccine dose prior to the outbreak, and 3.9% (89 cases) have received two prior measles vaccine doses. In 117 cases (5.2%), vaccination status could not be validated against a vaccination record or via the national immunization registry. All the cases with missing vaccination status were adults aged over 18 years (mean age 35.6 ± 8.2 years, median age 35.8 years). The two study groups differed as to measles vaccination status. The unvaccinated fraction was higher in the epidemiologically linked case group at 87.8%, compared to 66.7% in the laboratory confirmed case group (OR = 2.77, 95% CI 2.15–3.56, *p* = 0.0001). Accordingly, the fraction of cases with at least one prior measles vaccine dose (or more) was higher in the laboratory confirmed case group (22.6% vs. 9.6% in epidemiologically linked cases). The missing vaccination status fraction was higher in laboratory confirmed cases (10.8% vs. 2.6% in epidemiologically linked cases).

The multivariate analysis included a multiple logistic regression model for the dependent variable, defined as “measles laboratory confirmation” (Table 3). The multivariate model was analyzed independently for four age groups, since vaccination status is highly correlated with age (number of measles vaccine doses according to the vaccination schedule). The age groups were under 1 year (under the age for the first measles vaccine dose), 1–5 years (scheduled to receive the first vaccine dose at 1 year of age), 6–17 years (scheduled to receive the second vaccine dose at 6 years of age), and 18 years and above. The model variables included age, gender, place of residence, measles cases in the household, month of disease onset, measles vaccination status, and the place of medical treatment. After model adjustment, the variables that were significantly associated with higher odds for laboratory confirmation in the study groups included the month of disease onset (late), additional cases in the household (single case), place of medical treatment (hospital, either emergency department visit or hospitalization) and vaccination status (at least one vaccine dose).

Table 4 presents laboratory confirmed cases (*n* = 716) across three laboratory test groups (RT-PCR, serology for measles IgM and IgG, and RT-PCR and serology combination). Most laboratory confirmed cases (420, 58.7%) underwent real-time PCR tests. Serological tests were used in 189 (26.4%) of cases, and a combination of RT-PCR and serology in 107 cases (14.9%).

**Figure 3:**
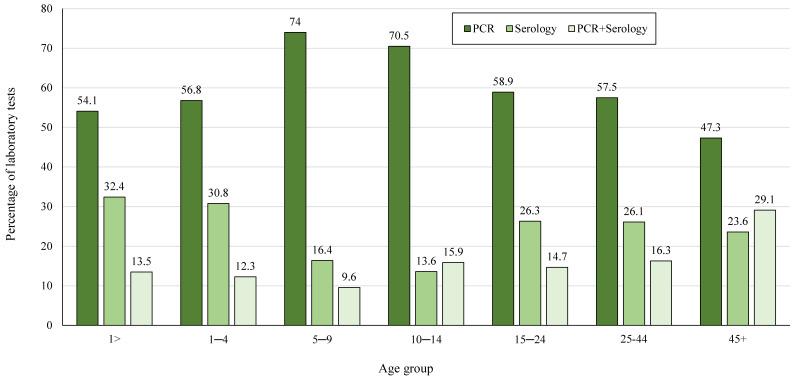
The group of laboratory confirmed measles cases (*n* = 716), according to the type of the laboratory test used, and according to seven age groups.

The laboratory confirmed measles cases were further analyzed according to the type of laboratory test used and the affected age groups (Figure 3). The distribution of type of tests used differed between age groups; over 70% of the cases in the groups of children aged 5–9 years and 10–14 years, compared to about 50% or less in the other age groups, were tested for using RT-PCR. The combined group of measles cases in children aged 5–14 years was compared to all the other age groups combined. The likelihood of RT-PCR was significantly higher in children aged 5–14 years (72.6% vs. 55.8%, OR = 2.1, 95% CI 1.34–3.37, *p* = 0.0001).

**Figure 4:**
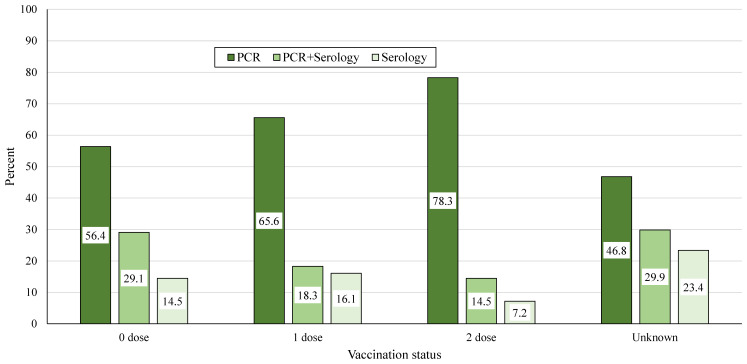
The group of laboratory confirmed measles cases (*n* = 716) according to the type of laboratory test used for diagnostics, and according to measles vaccination status.

Further analysis of the laboratory confirmed cases included laboratory test type and vaccination status (Figure 4). Measles cases with one or more prior vaccine doses were more likely to have been identified using RT-PCR (one vaccine dose 65.6%, two vaccine doses 78.3%), compared to 56.4% and 46.8% in unvaccinated cases and cases with unknown vaccination status, respectively. The group of measles cases with one or more previous vaccine doses was compared to the combined groups of unvaccinated cases and those with unknown vaccination status. The likelihood of RT-PCR was significantly higher in measles cases with one or more previous vaccine doses (71% vs. 55.1%, OR = 2.0, 95% CI 1.35–2.98, *p* = 0.0001).

According to the multivariate analysis model, young children aged one to five years had the greatest likelihood of being in the epidemiologically linked group (94.3%), having additional cases in the household, having been treated in community clinics, residing in dense urban neighborhoods, and having their cases reported during the peak of the outbreak. The model showed that the greatest likelihood of being in the laboratory confirmed group (80%) occurred for cases in patients older than 18 years, those treated in the hospital or emergency department, and those who did not have additional measles cases in the household.

## 4. Discussion

The current study portrays a comprehensive retrospective analysis of all measles cases (*n* = 2254) notified during a large outbreak in the Jerusalem district in 2018–2019. According to the public health guidelines, the measles outbreak cases were confirmed either using laboratory tests or by epidemiological linkage [13,14,15]. This study aimed to describe and assess measles case ascertainment by comparison of two study groups: the laboratory confirmed (*n* = 716 measles cases) and the epidemiologically confirmed (*n* = 1538 measles cases) cases. The main findings indicate significant differences between the two groups. These differences reflect the likelihood of measles laboratory confirmation versus epidemiological confirmation in a large outbreak setting. In the multivariate analysis model designed for measles laboratory confirmation, the associated variables included age, period of disease onset, additional measles cases in the household, place of medical treatment, and vaccination status prior to the outbreak onset. The laboratory method type (RT-PCR, serology, and combination) was associated with age group and vaccination status.

The peak incidence rates in the national measles outbreak (2018–2019) were observed in under-vaccinated communities (mainly Jewish ultra-orthodox) in the Jerusalem district [21]. Recurrent vaccine preventable disease outbreaks (e.g., measles, mumps, pertussis) had previously emerged, occasionally associated with international transmission (Europe, UK, and the US) [21,29]. The outbreak containment approach was based on the recommended activities of rapid case identification and isolation, mass vaccination of susceptible individuals, post exposure immunoglobulin in susceptible high-risk persons, and sustaining epidemiological and laboratory competencies for case confirmation [18,31]. The standard clinical case definition used in the outbreak comprised a generalized maculopapular rash, fever (≥38.3), and either a cough, coryza, or conjunctivitis [14,32]. The laboratory confirmation included RT-PCR, serological tests of measles IgM and IgG, and a combination of both [19,23,25]. A previous study in the US [32] demonstrated that the overall sensitivity of the measles clinical case definition is generally high, while the positive predictive value is intensely associated with measles incidence rates, being more accurate in higher incidence rate settings (of >171 per 100,000 population). The overall incidence rate of measles in the Jerusalem outbreak was 176 per 100,000 population, and hence it is plausible that the validity of the standard clinical case definition was appropriate [17].

The estimated number of measles cases globally increased considerably in 2022 compared to 2020 and 2021, and continued increasing during 2023 [1,33]. Recently, in December 2023, the WHO European Region issued an alert advocating for urgent action due to a thirty-fold rise in measles cases in the region (case notifications between January and October 2023). Clearly, measles outbreak containment is an extremely relevant public health challenge today [34]. The pause in progression towards the measles elimination goal might be attributed to the consequences of the COVID-19 pandemic on health systems worldwide. This led specifically to the disruption of routine childhood vaccination programs and an accompanying decline in childhood vaccination coverage rates, including the measles vaccines [1,35,36,37]. The Immunization Agenda 2021–2030 uses measles both as an indicator of the strength of the immunization system and as a tracer of health system capacity to deliver essential childhood vaccines [1]. Even in countries with a high overall immunization coverage, such as Israel, challenges exist. Such challenges include specific population groups with frequently delayed or incomplete childhood vaccinations (including measles vaccinations) [18,19,20,21,29]. Additionally, increasing globalization and international travel enables the rapid transmission of measles and further propagation of outbreaks in under-vaccinated communities in different countries [38,39,40,41]. While vaccination campaigns during outbreaks are traditionally successful [18], the sustainability of immunization coverage rates is less so [42].

Measles infection can occur at any age; however, the greatest disease burden globally is among children less than five years of age [3,10]. In this study, over three quarters (75.5%) of the measles cases were observed in patients less than fifteen years of age, and almost half (49.2%) were seen in patients less than five years of age. Notably, regarding the distribution of measles case ascertainment, the group of epidemiologically linked cases was significantly younger (median age 4.6 years) compared to the group of laboratory confirmed cases (median age 9.2 years). According to our multivariate analysis model, the strongest likelihood of being in the epidemiologically linked group (94.3%) was in measles cases with the following characteristics: children aged 1 to 5 years; additional cases in the household; treated in community clinics; and residing in a dense urban neighborhood. These cases mainly occurred during the peak of the measles outbreak, with widespread infection transmission via households, childcare facilities, kindergartens, and schools.

The highest incidence rates of measles in the outbreak were observed in infants less than one year old [17]. The measles cases in infants (*n* = 425) comprised about a fifth of the total study population, and were equally distributed between the epidemiologically linked group and the laboratory confirmed group. Notably, 84.7% of the infants under one year old were aged between 6 and 12 months. Measles vaccines in the national immunization program are scheduled at ages one and six. Therefore, infants less than one year of age are not yet eligible for vaccination [29]. Similarly, during the national measles outbreak in Israel in 2018–2019, the Tel Aviv district received notifications of 413 cases, with 100 (24%) of them being in infants under one year [43]. A concurrent measles outbreak occurred in New York (September 2018–July 2019) with most cases (93.4%, *n* = 649) observed in the Orthodox Jewish community, and 85.8% of the patients being unvaccinated [38]. Most cases (81.2%) were observed in patients younger than 18 years of age; the median age was 3 years [38]. In the New York outbreak, the measles cases in infants less than one year of age (*n* = 102) comprised 15.7% of all cases, and 72.5% of these infants were aged 6 to 12 months [38]. The overrepresentation of young infants in the outbreak (especially those aged 6–12 months) correlates well with the results of a seroprevalence study performed in Israel, before the 2018–2019 measles outbreak [25]. The overall seropositivity rate for measles was 90.7%. The lowest seropositivity rate was in infants aged 6–11 months (3.8%). The rates in infants under 6 months, in children 1–4 years old and in children 5–9 years old were 48.9%, 90.7%, and 96.1%, respectively [25]. Similarly, in a study on measles humoral immunity in infants under 12 months of age in Canada, most infants were susceptible to measles by the age of 3 months [44].

Laboratory diagnosis of measles may include viral isolation, identification of measles antigen or RNA in infected tissues (and nucleotide sequencing), or demonstration of a significant serologic response to measles virus with the detection of specific IgM [6]. Specifically, when aiming for global measles elimination, it is essential to maintain laboratory capabilities for rapid measles case ascertainment, enabling further control activities [31]. The public health laboratory infrastructure necessitates support both logistically and financially, as well as in the form of governmental commitment [2,9]. The methods used in our study for measles laboratory confirmation were RT-PCR, serological tests, and a combination of these two tests. The choice of laboratory test was significantly associated with the measles patients’ characteristics, e.g., age and vaccination status. Hence, the collaboration of epidemiological and laboratory public health capacities is essential.

The current study is subject to several limitations. It is an observational study, including all the notified measles cases in a measles outbreak in a well-defined geographic area (the Jerusalem district) and having validated data on their case characteristics and ascertainment method. Consequently, it might be challenging to make a generalization of the study findings. Distinctive socio-demographic factors, including age distribution, living conditions, household size, and the characteristics of the childcare facilities and population crowding, which significantly affect infectious disease transmission, may differ extensively between various settings and population groups and therefore make comparisons difficult. The local public health infrastructure and the epidemiological capabilities, as well as the laboratory competencies, may also differ considerably between outbreak settings.

## 5. Conclusions

Measles is a highly contagious viral disease; hence, sufficient herd immunity is essential to prevent transmission. Measles-containing vaccines have been available for decades and have contributed greatly to progress towards regional and global measles elimination [1,7,9]. Yet, this progress has been challenged due to inadequate vaccination rates in children, and associated worsening of these rates during the COVID-19 pandemic [1,35,36,37]. Measles cases notifications surged in 2022–2023, resulting in loss of elimination status in many countries [10]. The measles outbreak described in this study demonstrates the urgency of addressing vaccination gaps with appropriate outbreak prevention programs. The road to measles elimination needs to be paved with robust public health infrastructure, excellent field epidemiology for outbreak surveillance, investigation, and control, and laboratory proficiency.

## Figures and Tables

**Table 1:** General characteristics of the measles cases observed between 18 June and 31 May 2019 in the Jerusalem district (*n* = 2254 cases).

Variable	All *n* = 2254	Laboratory Confirmed Case *n* = 716	Epidemiologically-Linked to Confirmed Case *n* = 1538	Sig
Age median (years)	5.2	9.2	4.6	<0.001
Percentiles 25	1.4	1.2	1.5	
Percentiles75	14.8	28.5	10.3	
Age groups				
under 1 year	425 (18.9%)	148 (20.7%)	277 (18%)	NS
1–4	684 (30.3%)	148 (20.7%)	536 (34.9%)	0.0001
5–9	401 (17.7%)	74 (10.3%)	327 (21.3%)	0.0001
10–14	192 (8.5%)	44 (6.2%)	148 (9.6%)	0.006
15–24	238 (10.6%)	95 (13.3%)	143 (9.3%)	0.004
25–44	229 (10.1%)	152 (21.4%)	77 (5%)	0.0001
45+	85 (3.8%)	55 (7.7%)	30 (2%)	0.0001
Gender				
male	1306 (57.9%)	445 (62.2%)	861 (56%)	0.006
Female	948 (42.1%)	271 (37.8%)	677 (44%)	
Place of residence				
Jerusalem	1419 (62.9%)	436 (60.9%)	983 (63.9%)	0.001
other	835 (37.1%)	280 (39.1%)	555 (36.1%)	
Number of cases in the family median	1	1	2	<0.001
Percentiles 25	1	1	1	
Percentiles 75	3.25	2	5	
Cases in the household				
1 case	1172 (52%)	519 (72.5%)	653 (42.5%)	0.001
2 cases or more	1082 (48%)	197 (27.6%)	885 (57.5%)	
Period of disease onset (month)				
6/2018–10/2018	1037 (46%)	248 (34.6%)	789 (51.3%)	0.0001
11/2018–5/2019	1217 (54%)	468 (65.4%)	749 (48.7%)	
Place of daily stay				
At home	311 (13.8%)	84 (11.7%)	227 (14.8%)	NS
In a setting	1668 (74%)	548 (76.5%)	1120 (72.8%)	
Unknown	275 (12.2%)	84 (11.7%)	191 (12.4%)	

NS = Not Significant.

**Table 2:** Clinical characteristics and vaccination status of measles cases reported between June 2018 and 31 May 2019, Jerusalem district (*n* = 2254).

Variable	All *n* = 2254	Laboratory Confirmed *n* = 716	Epidemiologically-Linked *n* = 1538	Sig
Clinical signs				
fever (38.3 °C or above)	2170 (96.3%)	674 (94.1%)	1496 (97.3%)	0.0001
maculopapular rash	2100 (93.2%)	669 (93.4%)	1431 (93%)	NS
Coryza	1809 (80.3%)	555 (77.5%)	1254 (81.5%)	0.026
Cough	1897 (84.2%)	593 (82.8%)	1304 (84.8%)	NS
Conjunctivitis	1728 (76.7%)	527 (73.6%)	1201 (78.1%)	0.019
Pneumonia	154 (6.8%)	80 (11.2%)	74 (4.8%)	0.0001
Otitis	142 (6.3%)	65 (9.1%)	77 (5%)	0.0001
Place of treatment				
Community clinic	1849 (82%)	461 (64.4%)	1388 (90.2%)	0.0001
Emergency department	215 (9.5%)	125 (17.4%)	90 (5.9%)	0.0001
Hospitalization	190 (8.4%)	130 (18.2%)	60 (3.9%)	0.0001
Vaccination				
Unvaccinated (0 doses)	1828 (81.1%)	477 (66.7%)	1351 (87.8%)	0.0001
1 measles vaccine dose	220 (9.8%)	93 (13%)	127 (8.3%)	0.0001
2 measles vaccine doses.	89 (3.9%)	69 (9.6%)	20 (1.3%)	0.0001
Unknown	117 (5.2%)	77 (10.8%)	40 (2.6%)	0.0001

NS = Not Significant.

**Table 3:** Multivariate logistic regression analysis for laboratory confirmation by age group, measles cases from June 2018–31 May 2019, Jerusalem district (*n* = 2254).

	<1 Year *n* = 425		1–5 Year *n* = 787		6–17 Year *n* = 573		18+ Year *n* = 469	
	Adjusted OR (95% CI)	Sig	Adjusted OR (95% CI)	Sig	Adjusted OR (95% CI)	Sig	Adjusted OR (95% CI)	Sig
								
Age (month)	0.9 (0.8–1.005)	NS	0.99 (0.98–1.0)	NS	1 (0.99–1.00)	NS	1.002 (1.001–1.004)	0.012
Gender								
Male	1		1		1		1	
Female	0.95 (0.6–1.5)	NS	0.7 (0.47–1.03)	NS	0.48 (0.3–0.8)	0.002	1.3 (0.9–2.1)	NS
Place of residence								
Jerusalem	1		1		1		1	
Other	1.1 (0.7–1.7)	NS	1.8 (1.2–2.7)	0.002	1.3 (0.8–2.1)	NS	1.4 (0.92–2.3)	0.06
Cases in the household								
1 case	1.7 (1.03–2.96)	0.039	2.9 (1.9–4.4)	0.001	2.8 (1.8–4.5)	0.001	2.4 (1.5–3.9)	0.001
2 cases or more	1		1		1		1	
Period of disease onset (month)								
06/2018–10/2018	1		1		1		1	
11/2018–05/2019	1.5 (0.97–2.3)	NS	1.02 (0.69–1.5)	NS	2.1 (1.3–3.3)	0.001	1.5 (0.9–2.4)	NS
Vaccination								
0 doses	----		1		1		1	
1 measles vaccine dose	----		1.3 (0.77–2.1)	NS	3.3 (1.5–7.6)	0.004	0.9 (0.5–1.6)	NS
2 measles vaccine doses	----		----		5.3 (2–14.5)	0.001	4.6 (2.2–9.7)	0.001
Unknown	----		----		----		1.25 (0.7–2.1)	NS
Place of treatment								
Community clinic	1		1		1		1	
Emergency department	3.2 (1.8–5.9)	0.001	4.1 (2.1–7.89)	0.001	2.2 (0.8–6.1)	NS	2.8 (1.6–4.9)	0.001
Hospitalization	4.9 (2.6–8.98)	0.001	5.7 (2.9–10.8)	0.001	5.1 (1.3–19.3)	0.018	6.3 (3.1–12.9)	0.001

NS = Not Significant.

**Table 4:** General characteristics of laboratory confirmed measles cases from June 2018–31 May 2019, Jerusalem district (*n* = 716 cases).

Variable	PCR *n* = 420	Serology *n* = 189	PCR + Serology *n* = 107	Sig
Age median (years)	9.2	5.9	18.1	0.04
Percentiles 25	1.2	0.99	1.5	
				
75	26.3	28.3	36.8	
Age groups				
under 1 year	80 (19%)	48 (25.4%)	20 (18.7%)	NS
1–4	84 (20%)	45 (23.8%)	18 (16.8%)	NS
5–9	54 (12.9%)	12 (6.3%)	7 (6.5%)	0.01
10–14	31 (7.4%)	6 (3.2%)	7 (6.5%)	NS
15–24	56 (13.3%)	25 (13.2%)	14 (13.1%)	NS
25–44	89 (21.2%)	40 (21.2%)	25 (23.4%)	NS
45+	26 (6.2%)	13 (6.9%)	16 (15%)	0.05
Gender				
Male	264 (62.9%)	115 (60.8%)	66 (61.7%)	NS
Female	156 (37.1%)	74 (39.2%)	41 (38.3%)	
Place of residence				
Jerusalem	247 (58.8%)	116 (61.4%)	72 (67.3%)	NS
Other	173 (41.2%)	73 (38.6%)	35 (32.7%)	
Number of cases in the family				
				
1 case	297 (70.7%)	140 (74.1%)	82 (76.6%)	NS
2 cases or more	123 (29.3%)	49 (25.9%)	25 (23.4%)	
Period of disease onset (month)				
6/2018–10/2018	109 (26%)	85 (45%)	54 (50.5%)	0.001
11/2018–5/2019	311 (74%)	104 (55%)	53 (49.5%)	
Place of daily stay				
At home	48 (11.4%)	26 (13.8%)	10 (9.3%)	0.054
In a setting	329 (78.3%)	131 (69.3%)	88 (82.2%)	
Unknown	43 (10.2%)	32 (16.9%)	9 (8.4%)	

NS = Not Significant.

## Data Availability

The data presented in this study are available upon request from the corresponding author and subjected to the obligatory legal restrictions required by Israel’s Ministry of Health regarding personal health data confidentiality.

## References

[1] Minta A.A., Ferrari M., Antoni S., Portnoy A., Sbarra A., Lambert B., Hatcher C., Hsu C.H., Ho L.L., Steulet C. (2023). Progress toward Measles Elimination—Worldwide, 2000–2022. MMWR Morb. Mortal Wkl. Rep..

[2] Dunn J.J., Baldanti F., Puchhammer E., Panning M., Perez O., Harvala H. (2020). Pan American Society for Clinical Virology (PASCV) Clinical Practice and Public Policy Committee and the European Society for Clinical Virology (ESCV) Executive Committee. Measles is Back—Considerations for laboratory diagnosis. J. Clin. Virol..

[3] Hübschen J.M., Gouandjika-Vasilache I., Dina J. (2022). Measles. Lancet.

[4] Duprex W.P., Dutch R.E. (2023). Paramyxoviruses: Pathogenesis, Vaccines, Antivirals, and Prototypes for Pandemic Preparedness. J. Infect Dis..

[5] Guerra F.M., Bolotin S., Lim G., Heffernan J., Deeks S.L., Li Y., Crowcroft N.S. (2017). The basic reproduction number (R0) of measles: A systematic review. Lancet Infect Dis..

[6] Misin A., Antonello R.M., Di Bella S., Campisciano G., Zanotta N., Giacobbe D.R., Comar M., Luzzati R. (2020). Measles: An Overview of a Re-Emerging Disease in Children and Immunocompromised Patients. Microorganisms.

[7] Winter A.K., Lambert B., Klein D., Klepac P., Papadopoulos T., Truelove S., Burgess C., Santos H., Knapp J.K., Reef S.E. (2022). Feasibility of measles and rubella vaccination programmes for disease elimination: A modelling study. Lancet Glob. Health.

[8] Gastañaduy P.A., Goodson J.L., Panagiotakopoulos L., Rota P.A., Orenstein W.A., Patel M. (2021). Measles in the 21st Century: Progress Toward Achieving and Sustaining Elimination. J. Infect. Dis..

[9] Moss W.J., Shendale S., Lindstrand A., O’Brien K.L., Turner N., Goodman T., Kretsinger K., SAGE Working Group on Measles and Rubella Vaccines (2021). Measles and Rubella Eradication Feasibility Assessment Workshop Participants. Feasibility assessment of measles and rubella eradication. Vaccine.

[10] Measles and Rubella Surveillance Data. https://www.who.int/immunization/monitoring_surveillance/burden/vpd/surveillance_type/active/measles_monthlydata/en/.

[11] Strebel P.M., Orenstein W.A. (2019). Measles. N. Engl. J. Med..

[12] Nicolay N., Mirinaviciute G., Mollet T., Celentano L.P., Bacci S. (2020). Epidemiology of measles during the COVID-19 pandemic, a description of the surveillance data, 29 EU/EEA countries and the United Kingdom, January to May 2020. Eur. Surveill..

[13] Measles Outbreak Guide. https://www.who.int/publications/i/item/9789240052079.

[14] US Centers for Disease Control and Prevention National Notifiable Diseases Surveillance System (NNDSS). Measles/Rubeola 2013 Case Definition. Source: Office of Public Health Data, Surveillance, and Technology. https://ndc.services.cdc.gov/case-definitions/measles-2013/.

[15] World Health Organization Measles Outbreak Toolkit. Updated September 2022. https://www.who.int/emergencies/outbreak-toolkit/disease-outbreak-toolboxes/measles-outbreak-toolbox.

[16] Hübschen J.M., Bork S.M., Brown K.E., Mankertz A., Santibanez S., Ben Mamou M., Mulders M.N., Muller C.P. (2017). Challenges of measles and rubella laboratory diagnostic in the era of elimination. Clin. Microbiol. Infect..

[17] Stein-Zamir C., Abramson N., Shoob H. (2020). Notes from the Field: Large Measles Outbreak in Orthodox Jewish Communities—Jerusalem District, Israel, 2018–2019. MMWR Morb. Mortal Wkl. Rep..

[18] Stein-Zamir C., Abramson N., Edelstein N., Shoob H., Zentner G., Zimmerman D.R. (2019). Community-Oriented Epidemic Preparedness and Response to the Jerusalem 2018–2019 Measles Epidemic. Am. J. Public Health..

[19] Ben-Chetrit E., Oster Y., Jarjou’i A., Megged O., Lachish T., Cohen M.J., Stein-Zamir C., Ivgi H., Rivkin M., Milgrom Y. (2020). Measles-related hospitalizations and associated complications in Jerusalem, 2018–2019. Clin. Microbiol. Infect..

[20] Anis E., Haas E.J., Indenbaum V., Singer S.R., Warshavsky B., Rishpon S., Green M.S., Mendelson E., Grotto I., Kaliner E. (2021). A prolonged, nationwide measles outbreak despite very high vaccination coverage in Israel, 2018–2019. J. Infect..

[21] Stein-Zamir C., Levine H. (2021). The measles outbreak in Israel in 2018–2019: Lessons for COVID-19 pandemic. Hum. Vaccin. Immunother..

[22] Stein-Zamir C., Shoob H., Abramson D. (2023). Measles clinical presentation, hospitalization and vaccination status among children in a community-wide outbreak. Vaccine.

[23] Mulders M. WHO Manual for the Laboratory-based Surveillance of Measles, Rubella, and Congenital Rubella Syndrome. Third edition, June 2018. https://www.technet-21.org/en/topics/programme-management/manual-for-the-laboratory-based-surveillance-of-measles-rubella-and-congenital-rubella-syndrome/manual-for-the-laboratory-based-surveillance-of-measles-rubella-and-congenital-rubella-syndrome.

[24] Hummel K.B., Lowe L., Bellini W.J., Rota P.A. (2006). Development of quantitative gene-specific real-time RT-PCR assays for the detection of measles virus in clinical specimens. J. Virol. Methods.

[25] Bassal R., Indenbaum V., Pando R., Levin T., Shinar E., Amichay D., Barak M., Ben-Dor A., Haim A.B., Mendelson E. (2021). Seropositivity of measles antibodies in the Israeli population prior to the nationwide 2018–2019 outbreak. Hum. Vaccin. Immunother..

[26] Brown K.E., Rota P.A., Goodson J.L., Williams D., Abernathy E., Takeda M., Mulders M.N. (2019). Genetic Characterization of Measles and Rubella Viruses Detected Through Global Measles and Rubella Elimination Surveillance, 2016–2018. MMWR Morb. Mortal. Wkl. Rep..

[27] Bucris E., Indenbaum V., Azar R., Erster O., Haas E., Mendelson E., Zuckerman N.S. (2021). Direct sequencing of measles virus complete genomes in the midst of a large-scale outbreak. PLoS ONE.

[28] World Health Organization (2022). Update: Circulation of active genotypes of measles virus and recommendations for use of sequence analysis to monitor viral transmission. Wkly. Epidemiol. Rec..

[29] Stein-Zamir C., Israeli A., Grotto I. (2021). Immunization registry as a digital assessment tool during outbreaks. Clin. Microbiol. Infect..

[30] Stein-Zamir C., Zentner G., Tallen-Gozani E., Grotto I. (2010). The Israel National Immunization Registry. Isr. Med. Assoc. J..

[31] Gastañaduy P.A., Banerjee E., DeBolt C., Bravo-Alcántara P., Samad S.A., Pastor D., Rota P.A., Patel M., Crowcroft N.S., Durrheim D.N. (2018). Public health responses during measles outbreaks in elimination settings: Strategies and challenge. Hum. Vaccin. Immunother..

[32] Hutchins S.S., Papania M.J., Amler R., Maes E.F., Grabowsky M., Bromberg K., Glasglow V., Speed T., Bellini W.J., Orenstein W.A. (2004). Evaluation of the measles clinical case definition. J. Infect. Dis..

[33] World Health Organization. https://www.who.int/teams/immunization-vaccines-and-biologicals/immunization-analysis-and-insights/surveillance/monitoring/provisional-monthly-measles-and-rubella-data.

[34] World Health Organization Home/News/A 30-Fold Rise of Measles Cases in 2023 in the WHO European Region Warrants Urgent Action. 14 December 2023. https://www.who.int/europe/news/item/14-12-2023-a-30-fold-rise-of-measles-cases-in-2023-in-the-who-european-region-warrants-urgent-action.

[35] Shmueli M., Lendner I., Ben-Shimol S. (2024). Effect of the COVID-19 pandemic on the pediatric infectious disease landscape. Eur. J. Pediatr..

[36] Maltezou H.C., Medic S., Cassimos D.C., Effraimidou E., Poland G.A. (2022). Decreasing routine vaccination rates in children in the COVID-19 era. Vaccine.

[37] Locke J., Marinkovic A., Hamdy K., Balendra V., Sanyaolu A. (2023). Routine pediatric vaccinations during the COVID-19 pandemic: A review of the global impact. World J. Virol..

[38] Zucker J.R., Rosen J.B., Iwamoto M., Arciuolo R.J., Langdon-Embry M., Vora N.M., Rakeman J.L., Isaac B.M., Jean A., Asfaw M. (2020). Consequences of Undervaccination—Measles Outbreak, New York City, 2018–2019. N. Engl. J. Med..

[39] Feemster K.A., Szipszky C. (2020). Resurgence of measles in the United States: How did we get here?. Curr. Opin. Pediatr..

[40] Kauffmann F., Heffernan C., Meurice F., Ota M.O.C., Vetter V., Casabona G. (2021). Measles, mumps, rubella prevention: How can we do better?. Expert Rev. Vaccines.

[41] Ekezie W., Awwad S., Krauchenberg A., Karara N., Dembiński Ł., Grossman Z., Del Torso S., Dornbusch H.J., Neves A., Copley S. (2022). Access to Vaccination among Disadvantaged, Isolated and Difficult-to-Reach Communities in the WHO European Region: A Systematic Review. Vaccines.

[42] Paret M., Trillo R., Lighter J., Youngster I., Ratner A.J., Pellett Madan R. (2022). Poor Uptake of MMR Vaccine 1-year Post-Measles Outbreak: New York City and Israel. J. Pediatr. Infect Dis. Soc..

[43] Salama M., Indenbaum V., Nuss N., Savion M., Mor Z., Amitai Z., Yoabob I., Sheffer R. (2021). A Measles Outbreak in the Tel Aviv District, Israel, 2018–2019. Clin. Infect Dis..

[44] Science M., Savage R., Severini A., McLachlan E., Hughes S.L., Arnold C., Richardson S., Crowcroft N., Deeks S., Halperin S. (2019). Measles Antibody Levels in Young Infants. Pediatrics.

